# The clinical significance of the T2-FLAIR mismatch sign in grade II and III gliomas: a population-based study

**DOI:** 10.1186/s12885-020-06951-w

**Published:** 2020-05-20

**Authors:** Alba Corell, Sandra Ferreyra Vega, Nickoleta Hoefling, Louise Carstam, Anja Smits, Thomas Olsson Bontell, Isabella M. Björkman-Burtscher, Helena Carén, Asgeir Store Jakola

**Affiliations:** 1 Department of Neurosurgery, Sahlgrenska University Hospital, Gothenburg, Sweden; 2 Department of Clinical Neuroscience, Institute of Neuroscience and Physiology, University of Gothenburg, Sahlgrenska Academy, Gothenburg, Sweden; 3 Department of Radiology, Sahlgrenska University Hospital, Gothenburg, Sweden; 4 Department of Radiology, Institute of Clinical Sciences, Sahlgrenska Academy, University of Gothenburg, Gothenburg, Sweden; 5 Department of Neuroscience, Neurology, Uppsala University, Uppsala, Sweden; 6 Department of Clinical Pathology and Cytology, Sahlgrenska University Hospital, Gothenburg, Sweden; 7 Department of Physiology, Institute of Neuroscience and Physiology, University of Gothenburg, Sahlgrenska Academy, Gothenburg, Sweden; 8 Sahlgrenska Cancer Center, Department of Laboratory Medicine, Institute of Biomedicine, Sahlgrenska Academy, University of Gothenburg, Gothenburg, Sweden; 9 Department of Neuromedicine and Movement Science, NTNU, Trondheim, Norway

**Keywords:** Astrocytoma, Oligodendroglioma, Biomarkers, Decision making, prognosis

## Abstract

**Background:**

The T2-FLAIR mismatch sign is an imaging finding highly suggestive of isocitrate dehydrogenase mutated (*IDH-*mut) 1p19q non-codeleted (non-codel) gliomas (astrocytomas). In previous studies, it has shown excellent specificity but limited sensitivity for *IDH*-mut astrocytomas. Whether the mismatch sign is a marker of a clinically relevant subtype of *IDH-*mut astrocytomas is unknown.

**Methods:**

We included histopathologically verified supratentorial lower-grade gliomas (LGG) WHO grade II-III retrospectively during the period 2010–2016. In the period 2017–2018, patients with suspected LGG radiologically were prospectively included, and in this cohort other diagnoses than glioma could occur. Clinical, radiological and molecular data were collected. For clinical evaluation we included all patients with *IDH*-mut astrocytomas. In the 2010–2016 cohort DNA methylation analysis with Infinium MethylationEPIC BeadChip (Illumina) was performed for patients with an *IDH*-mut astrocytoma with available tissue. We aimed to examine the association of the T2-FLAIR mismatch sign with clinical factors and outcomes. Additionally, we evaluated the diagnostic reliability of the mismatch sign and its relation to methylation profiles.

**Results:**

Out of 215 patients with LGG, 135 had known *IDH-*mutation and 1p19q codeletion status. Fifty patients had an *IDH*-mut astrocytoma and 12 of these (24.0%) showed a mismatch sign. The sensitivity and specificity of the mismatch sign for *IDH*-mut detection were 26.4 and 97.6%, respectively. There were no differences between patients with an *IDH*-mut astrocytoma with or without mismatch sign when grouped according to T2-FLAIR mismatch sign with respect to baseline characteristics, clinical outcomes and methylation profiles. The overall interrater agreement between neuroradiologist and clinical neurosurgeons for the T2-FLAIR mismatch sign was significant when all 215 MRI examination assessed (κ = 0.77, *p* < 0.001, *N* = 215).

**Conclusion:**

The T2-FLAIR mismatch sign in patients with an *IDH*-mut astrocytoma is not associated with clinical presentation or outcome. It seems unlikely that the *IDH*-mut astrocytomas with mismatch sign represent a specific subentity. Finally, we have validated that the T2-FLAIR mismatch sign is a reliable and specific marker of *IDH*-mut astrocytomas.

## Background

Lower-grade gliomas (LGG) are intra-axial neoplasms of the brain, including WHO grade II and III astrocytomas and oligodendrogliomas according to the WHO 2016 classification and The Cancer Genome Atlas Research Network [1, 2]. These subgroups are based upon determination of mutation in the isocitrate dehydrogenase genes 1 or 2 (*IDH1* and *IDH2*), and 1p19q codeletion status [3, 4]. In addition to providing tumor classification, these markers also provide important prognostic information [1, 2, 4–6].

Biomarkers are the fundamental keystones of personalized management strategies, but the traditional biomarkers analyzed in tumor tissue come into play only after the surgical procedure. Thus, in the neurosurgical decision making, image-based biomarkers are of particular interest to identify relevant subgroups of patients. The newly described imaging feature of T2-FLAIR (fluid attenuation inversion recovery) mismatch sign has gained increased attention, since it is a widely available and simple potential imaging marker to predict *IDH*-mutated *(IDH*-mut*)* 1p19q non-codeleted (non-codel) gliomas (astrocytoma) with high specificity [7, 8]. The T2-FLAIR mismatch sign (further also referred to as mismatch sign) is characterized by a hyperintense signal on T2-weighted sequences and a hypointense signal on FLAIR sequences with a hyperintense peripheral rim, see Supplementary Figs. 1, 2 and 3 as examples.

DNA methylation analysis is in the frontline of diagnostic technology in gliomas [9–11]. The tumors showing a mismatch sign on MRI differ radiologically from gliomas without the mismatch sign with their distinct features. This raises questions regarding underlying biology. Studies so far, however, have not indicated that this radiological marker is reflected by a specific biological signature [7, 12].

An important question remains whether *IDH*-mut astrocytomas with or without mismatch sign reflect differences of clinical relevance, including resectability, based on the T2-weighted appearance being homogeneous and well demarcated or not. For instance, if *IDH*-mut astrocytomas with mismatch sign can be depicted by neuroimaging as more “resectable”, this tool would provide important information prior to surgery, where the extent of resection is of particular importance for patients with *IDH*-mut astrocytomas [13, 14]. In addition, institutional experience from neurosurgeons over the years has led to speculations about differences in texture in some tumors, being softer or more gelatinous and perhaps easier to remove. Since size, location and proximity to critical structures are of such importance in surgical decision-making, the identification of an imaging marker pointing towards factors affecting resection, could indeed alter surgical management, especially in complicated cases [15].

We aimed to evaluate clinical parameters including extent of resection with regard to mismatch sign. In addition, we analyzed if *IDH*-mut astrocytomas with mismatch sign had similar methylation profiles compared to samples without mismatch sign. Finally, we provide interrater variability between neurosurgeons and neuroradiologist and the sensitivity and specificity of the mismatch sign.

## Methods

All patients in the Västra Götaland region in Sweden with newly diagnosed primary intracranial intra-axial tumors are managed in a multidisciplinary team (MDT) with weekly conferences at the Sahlgrenska University Hospital. The neurosurgical department at the Sahlgrenska University Hospital in Gothenburg covers the population of approximately 1.7 million inhabitants.

**Fig. 1:**
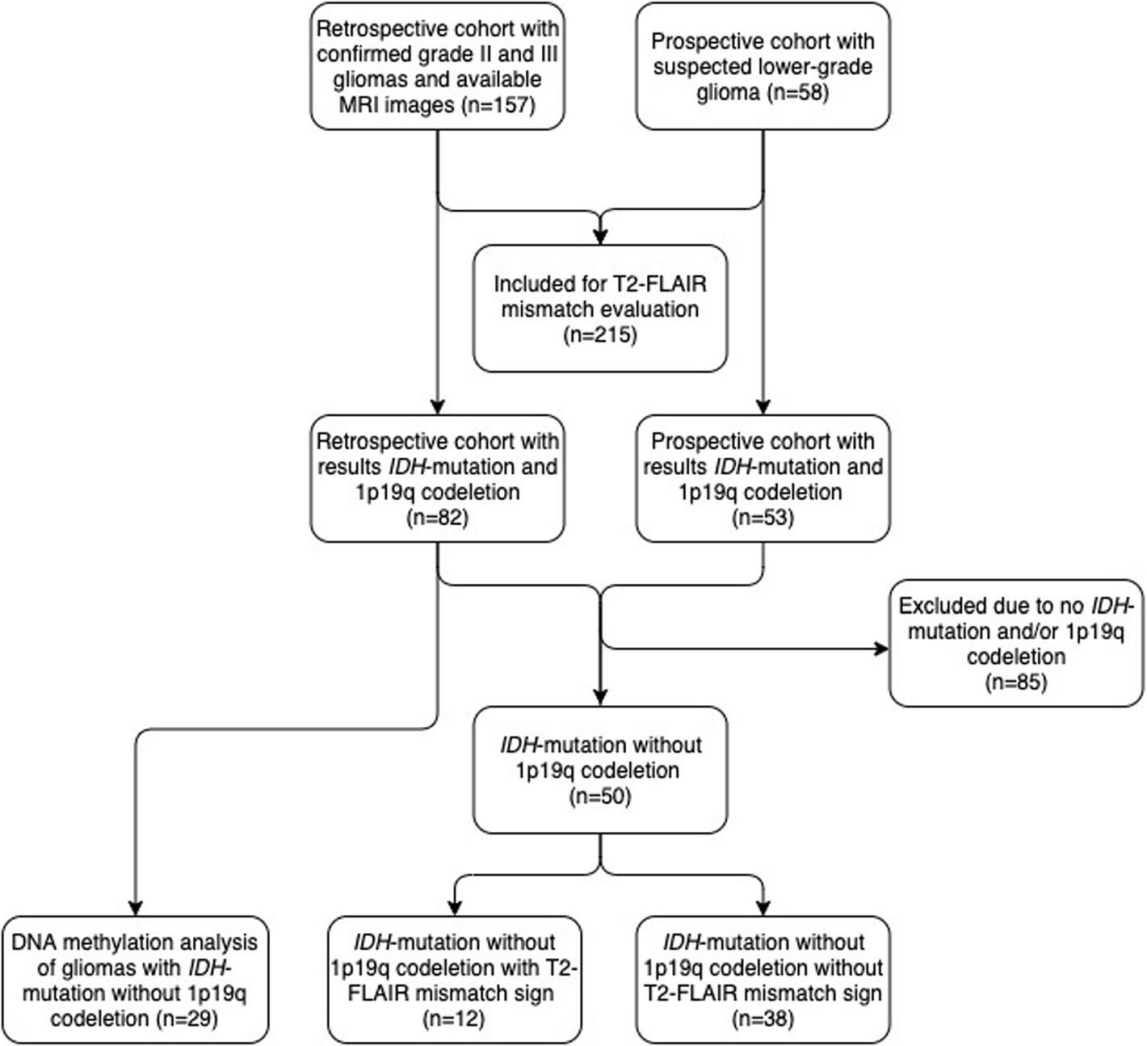
Flowchart of patient inclusion

Our cohort consists of two components; one retrospective cohort and one prospective. For patient selection see flowchart in Fig. 1. We performed a *retrospective* collection of clinical and radiological data between 2010 and 2016, searching operation logs and pathology database, thus covering all patients with a histopathological diagnosis of a supratentorial infiltrating WHO grade II or III glioma with magnetic resonance imaging (MRI) [1, 16].

In the years 2017 and 2018, we included patients *prospectively* based on a high suspicion of LGG grade II and III, referred either to the MDT conference or directly to the neurosurgical department. In principle, our cohort consists of lesions indicative of primary brain tumor with hyperintense signal in T2 weighted images, but with no significant contrast enhancement. In this cohort, other histopathological diagnoses may be encountered (e.g. other tumor subgroups or even non-neoplastic lesions). The rationale for “blindly” including these patients was to enable the evaluation of the mismatch sign in a group with rather similar MRI appearance, but also differential diagnoses of LGG, in contrast to most previous studies that used histopathology as selection [7, 17].

We used MRI images from all patients in the retro- and prospective cohorts to perform the interrater evaluation of the T2-FLAIR mismatch sign (*N* = 215). In further clinical evaluations, we analyzed the mismatch sign in patients with known *IDH*-mut gliomas without 1p19q codeletion from both cohorts (*N* = 50). The DNA methylation was performed in patients from the retrospective cohort with *IDH*-mut non-codel gliomas (*N* = 29).

Clinical variables such as patients’ age, sex, symptoms at diagnosis and Karnofsky functional status [18] were recorded. For basic radiological variables we included main lobe involved, tumor border (absent, mild/moderate or conspicuous) and eloquence [19]. We analyzed patients with *IDH*-mut astrocytomas divided into two groups, with or without mismatch sign.

### Image acquisition

MRI examinations reviewed in this project were performed at different hospitals as part of the clinical pre-operative work up in these tumor patients. MRI systems used for image acquisition included both 1.5 T and 3.0 T scanners from different vendors (GE Healthcare, US; Philips, The Netherlands; Siemens Healthcare, Germany) and with different software releases. 2D sequences with accepted slice thicknesses ≤5 mm were predominant. For T2-weighted sequences, median repetition time was 4000 milliseconds (ms) and median echo time was 100 ms. For FLAIR examinations, median repetition time was 9000 ms, median echo time was 122 ms, and median inversion time was 2500 ms. All scanners underwent regular maintenance by the vendors and sequences were optimized by the hospitals for clinical evaluation of brain lesions such as brain tumors.

### Image evaluation

Images were analyzed for: main lobe involved (frontal, temporal, parietal, occipital, insula), side (right, left, bilateral), border (absent, mild/moderate or conspicuous), eloquence [19], T2-FLAIR mismatch (yes/no), and size (volume by tumor segmentation).

### T2-FLAIR mismatch analysis

The MRI scans were evaluated for the T2-FLAIR mismatch sign as done by Patel et al [7]. Evaluation was independently performed by a neurosurgical resident (AC), a senior neurosurgeon (ASJ) and a board certified neuroradiologist (NH). In case of disagreement between the clinicians a consensus reading was performed between the neurosurgeons and interrater agreement was calculated. The consensus was then compared to the independent reading of the neuroradiologists (interrater agreement) and in case of disagreement, a final consensus reading was performed with a senior neuroradiologist (IBB).

### Tumor segmentation

Both T2 and FLAIR sequences were used for tumor volume segmentation, depending on which sequence the tumor was more clearly visible. FLAIR was often used in the preoperative MRI. Due to surgically-induced artifacts, T2 was sometimes preferred in the postoperative MRI and evaluation was done on a case-by-case basis. This selection was done up-front, and we did not segment both T2 and FLAIR sequences with later selection of the more “beneficial” one.

The tumor volume was evaluated by semi-automatic segmentation performed with the open-source software “3DSlicer”, version 4.6.2 [20]. For the segmentation of tumor volume, we used the tools “LevelTracingEffect”, “WandEffect”, “DrawEffect” and “PaintEffect” in the “Editor” module when appropriate. Tumor volumes were computed by the segmentation of hyperintensive areas on the T2 or FLAIR sequence on MRI examinations. Areas attributed to mainly edema without convincing signs of tumor invasion were excluded. In the gliomas with T2-FLAIR mismatch sign we used the outer margin of the peripheral rim on FLAIR images as the outer tumor border. Segmentation was performed by the neurosurgical resident (AC) with quality control in all cases from a senior neurosurgeon (ASJ) and neuroradiological expertise used in selected cases.

### DNA methylation array

DNA from formalin-fixed paraffin embedded (FFPE) tumors from patients included in the retrospective cohort was isolated with the QIAamp® DNA FFPE kit (Qiagen, Hilden, Germany) according to the supplier’s instructions with the addition of an extra digestion step with proteinase K overnight. DNA concentration was measured with the Qubit Fluorometer (Life technologies™, Carlsbad, CA, USA). Between 500 and 1000 ng DNA was bisulfite-converted with the EZ DNA methylation kit (D5001, Zymo Research, Irvine, CA, USA) and the methylation levels of restored bisulfite-converted DNA was determined with the Infinium MethylationEPIC BeadChip (Illumina®, San Diego, CA, USA) according to the protocols provided by the supplier.

Methylation analysis and normalization was performed as previously described [21]. Briefly, methylation data were processed with the statistical software R (version 3.6.1) using the Minfi [22] and ChAMP [22–24] packages. *IDH* mutational status was acquired using a published DNA methylation-based classifier [9]. 1p19q codeletion status was acquired through copy number variations inferred from the array. Correlation between the T2-FLAIR mismatch sign and DNA-methylation profiles was evaluated by unsupervised hierarchical clustering of the 5000 most variable CpG sites including only patients with *IDH*-mut non-codel gliomas (*N* = 29).

### Statistics

All statistical analyses were done with SPSS, version 24.0 (Chicago, IL, USA). Statistical significance level was set to *p* < 0.05 and all tests were two-sided. Central tendencies were presented as means ± standard deviation (SD), or median and first quartile (Q1) to third quartile (Q3) if skewed. Categorical data were analyzed with Pearson’s chi square test, but in 2 × 2 tables Fishers exact test was used when appropriate due to small sample. For continuous data independent sample t-test or Mann-Whitney U test were used as appropriate based upon data distribution. Interrater agreement between the clinical assessment by neurosurgeons and neuroradiologist for the presence or absence of the mismatch sign was assessed with Cohen’s kappa statistic (k) [25]. We considered > 0.6 to be substantial agreement, 0.41–0.6 moderate agreement, 0.21–0.4 fair agreement and ≤ 0.2 slight agreement [25]. To evaluate the interrater agreement, we used all patients in the retrospective and prospective cohort, regardless of molecular status. Finally, we present sensitivity and specificity for T2-FLAIR mismatch sign as a marker to identify *IDH*-mut astrocytomas.

### Ethics statement

This project was approved by the regional ethical committee in the region of Västra Götaland (DNR 1067–16 and DNR 363–17).

## Results

### Clinical factors and outcomes

Our patient cohort included retro- and prospectively 135 patients with available MRI images and known status of *IDH*-mutation and 1p19q codeletion. The *retrospective* part of the cohort included 82 patients with mean age of 45.0 years (SD 14.3) and 37 patients (45.1%) were females. The majority of this cohort underwent resection, as opposed to biopsy only (*N* = 77, 93.9%).

In the *prospective* part, we evaluated 58 patients with a *suspected* LGG. This included both neoplastic and non-neoplastic diagnoses, such as limbic encephalitis. Of these, 53 patients had known status of *IDH*-mutation and 1p19q codeletion. In this cohort, 22 patients were female (41.5%) and the mean age was 47.9 years (SD 15.7). Resection was the most common surgical treatment (*n* = 46, 86.8%). The most common histopathological diagnoses were WHO grade II or III astrocytoma (*N* = 27, 50.9%), oligodendroglioma (*N* = 21, 36.9%) and glioblastoma (*N* = 4, 7.5%). Other diagnoses included non-neoplastic lesions such as limbic encephalitis. In addition, one patient (1.9%) had other diagnosis (DNET). The mismatch sign was not present in any of the non-neoplastic diagnoses, or in the patient with DNET.

In total there were 50 patients with *IDH*-mut astrocytoma. These were grouped based upon presence of T2-FLAIR mismatch sign (*N* = 12, 24.0%) or absence (*N* = 38, 76.0%). In Table 1 we present comparison between these groups with respect to baseline characteristics, radiological variables and clinical outcome. The only significant difference was a more conspicuous tumor border in the group with mismatch sign (*p* = 0.02). There were no differences regarding tumor location, pre- and postoperative volumes, symptoms or type of surgery. Importantly, there were no differences between groups with respect to the extent of resection (87.9% with mismatch sign and 89.2% without mismatch sign, *p* = 0.91) or survival (median of 85 months in cohort with mismatch sign vs 65 months without mismatch sign, p = 0.91).

**Table 1:** Presentation and outcomes in patients diagnosed between 2010 and 2018 with *IDH*-mut astrocytomas (*N* = 50), presented in relation to the T2-FLAIR mismatch sign presence or absence

	Mismatch (***N*** = 12, 24.0%)	No mismatch (N = 38, 76.0%)	***P***-value
Age, years, mean (SD)	35.7 (12.6)	41.9 (14.4)	0.06
Female, n (%)	7 (58.3)	16 (42.1)	0.51
**Main lobe involved, n (%)**			
Frontal	7 (58.3)	17 (44.7)	0.51
Temporal	3 (25.0)	13 (34.2)	0.73
Parietal	2 (16.7)	7 (18.4)	1.00
Insula	0 (0.0)	1 (2.6)	1.00
**Image findings, n (%)**			
Right side	6 (50.0)	15 (39.5)	0.74
Left side	6 (50.0)	23 (60.5)	0.74
Bilateral/midline	0 (0.0)	5 (13.2)	0.32
Conspicuous border	9 (75.0)	12 (31.6)	0.02
Eloquence	8 (66.7)	30 (78.9)	0.45
**Symptom at diagnosis**^a^**, n (%)**			
Asymptomatic	0 (0.0)	3 (7.9)	1.00
Seizure	9 (75.0)	28 (73.7)	1.00
ICP related	3 (25.0)	11 (28.9)	1.00
Deficit(s)	0 (0.0)	5 (13.2)	0.32
Language deficit	2 (16.7)	2 (5.3)	0.24
Visual deficit	2 (16.7)	4 (10.5)	0.62
Cognitive changes	3 (25.0)	8 (21.1)	1.00
Other symptoms	1 (8.3)	10 (26.3)	0.25
**Type of surgery, n (%)**			
Resection	11 (91.7)	37 (97.4)	0.43
**Volumetric measurements**			
Preoperative volume, ml, median (Q1-Q3)	47.4 (29.71–113.61) *N* = 11	63.3 (26.52–115.32) *N* = 37	0.91
Postoperative volume, ml, median (Q1-Q3)	5.9 (1.67–14.81) N = 11	5.0 (0.34–16.42) N = 37	0.99
Extent of resection, median % (Q1-Q3)	87.9 (73.60–96.63) N = 11	89.2 (48.50–99.75) N = 37	0.91
**WHO grade, n (%)**			
WHO grade II	5 (41.7)	24 (63.2)	0.31
WHO grade III	6 (50.0)	13 (34.2)	0.50
WHO grade IV	1 (8.3)	1 (2.6)	0.43

^a^More than one symptom at presentation possible. Other symptoms included paresthesia, vertigo, dysphagia, among others

### Diagnostic properties

**Fig. 2:**
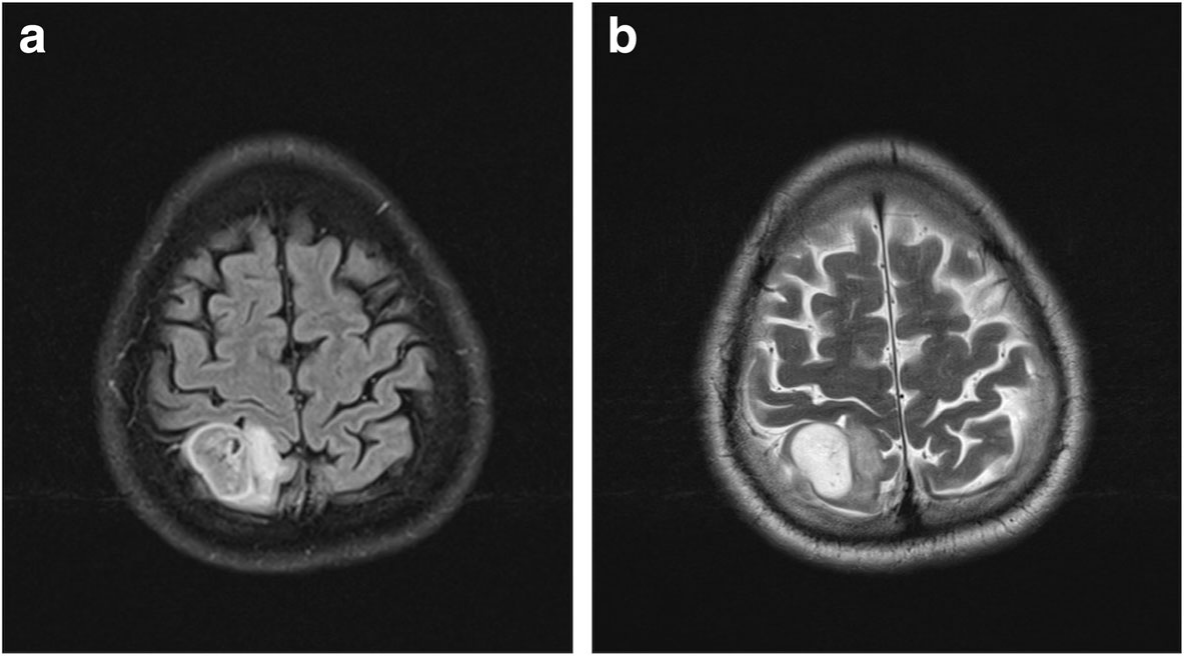
**a**-**b**: **a**) FLAIR sequence demonstrating a relative hypointense signal with the exception of a hyperintense peripheral rim. **b**) T2W sequence demonstrating homogenous hyperintensive signal with a conspicuous border. This glioma was considered to have a mismatch sign and was diagnosed with an *IDH*-mutated and 1p19q codeleted glioma (i.e. oligodendroglioma)

**Fig. 3:**
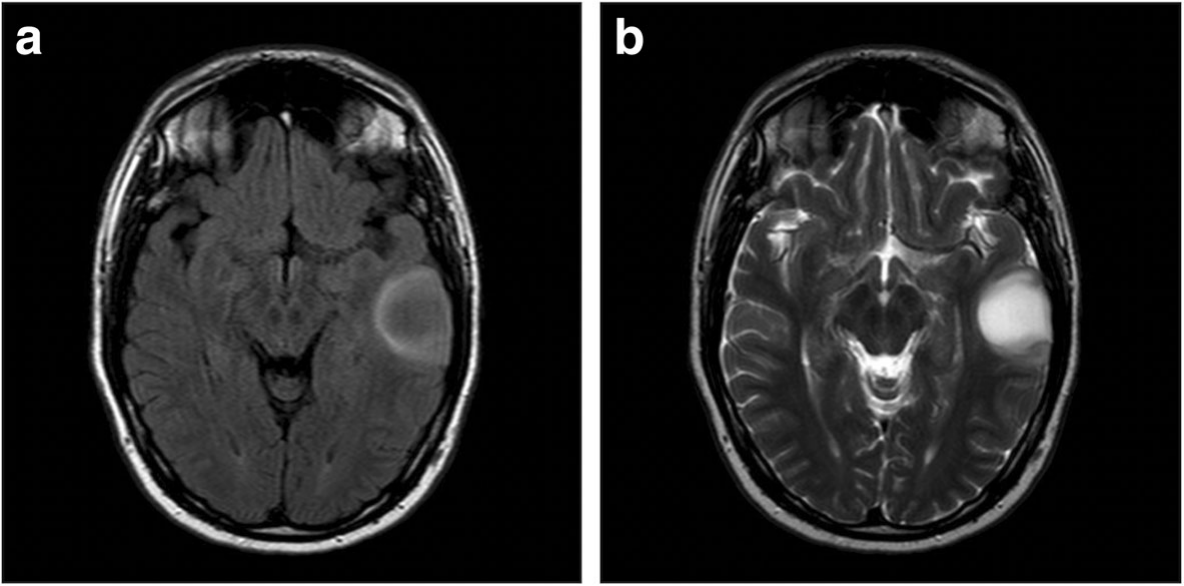
**a**-**b**: **a**) FLAIR sequence demonstrating a relative hypointense signal with the exception of a hyperintense peripheral rim. **b**) T2W sequence demonstrating homogenous hyperintensive signal with a conspicuous border. This glioma was considered to have a mismatch sign and was diagnosed with an *IDH*-mutated and 1p19q codeleted glioma (i.e. oligodendroglioma)

**Fig. 4:**
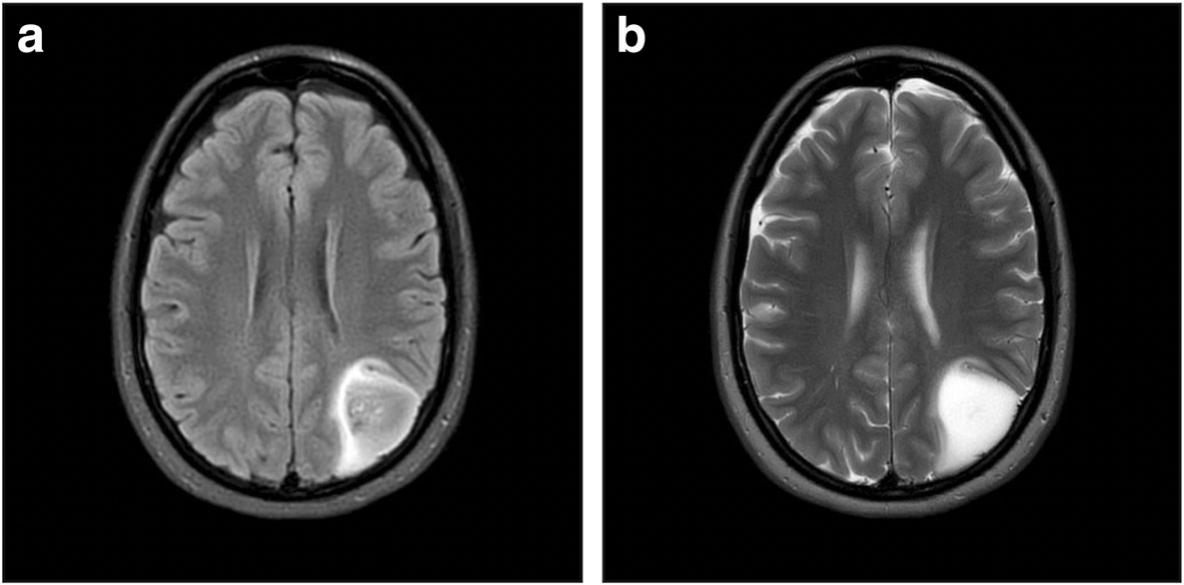
**a**-**b**: **a**) FLAIR sequence demonstrating a relative hypointense signal with the exception of a hyperintense peripheral rim. **b**) T2W sequence demonstrating homogenous hyperintensive signal with a conspicuous border. This glioma was considered to have a mismatch sign and the histopathological diagnosis was glioblastoma (i.e. astrocytoma WHO grade IV, *IDH*-mut)

Nine patients (10.9%) in the retrospective part of the cohort showed mismatch signs and all of them had *IDH*-mutated gliomas. There were no patients with a glioma with *IDH*-wild type and mismatch sign in the retrospective cohort. However, we identified two patients with 1p19q codeletion (codel) tumors (oligodendrogliomas) and mismatch sign (see Fig. 2a-b and 3a-b). In the prospective cohort, the lesion of 5 patients (8.6%) showed a mismatch sign, all of them being *IDH*-mut astrocytoma whereof one being WHO grade IV (glioblastoma, see Fig. 4a-b). One patient with a DNET did not show a positive T2-FLAIR mismatch sign.

In the joint cohort with known molecular status, including both *IDH*-mutation and 1p19q codeletion (*N* = 135), the specificity for *IDH*-mut astrocytomas was 97.6% and the sensitivity was 26.4%. The positive predictive value (PPV) was 85.7% and the negative predictive value (NPV) was 67.7%.

### Interrater agreement

**Fig. 5:**
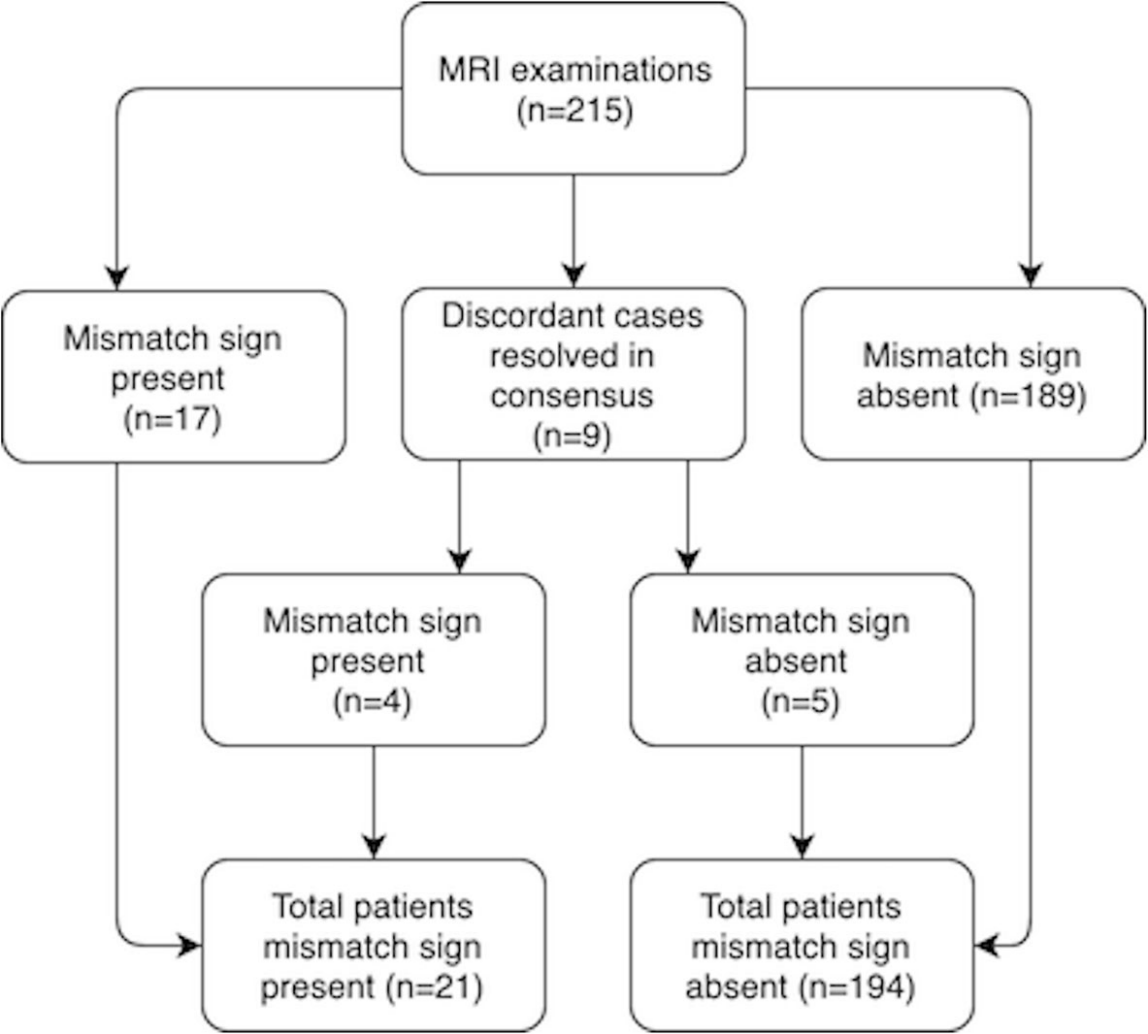
Overview over MRI mismatch sign assessment and agreement among raters

We evaluated 215 cases for the mismatch sign and it was found in 17 cases (7.9%), absent in 189 cases (87.9%), while 9 cases were discordant (4.2%), as demonstrated in Fig. 5. The total number of patients with mismatch sign was 21 (9.8%) after we reached consensus for the 9 discordant cases (discordant cases are presented in Supplementary material). The interrater agreement for the mismatch sign between clinical neurosurgeons was at a kappa value of 0.74 (*p* = 0.064), and between clinical neurosurgeons and neuroradiologist 0.77 (*p* < 0.001).

### Molecular markers and genome-wide DNA methylation array, retrospective cohort

**Fig. 6:**
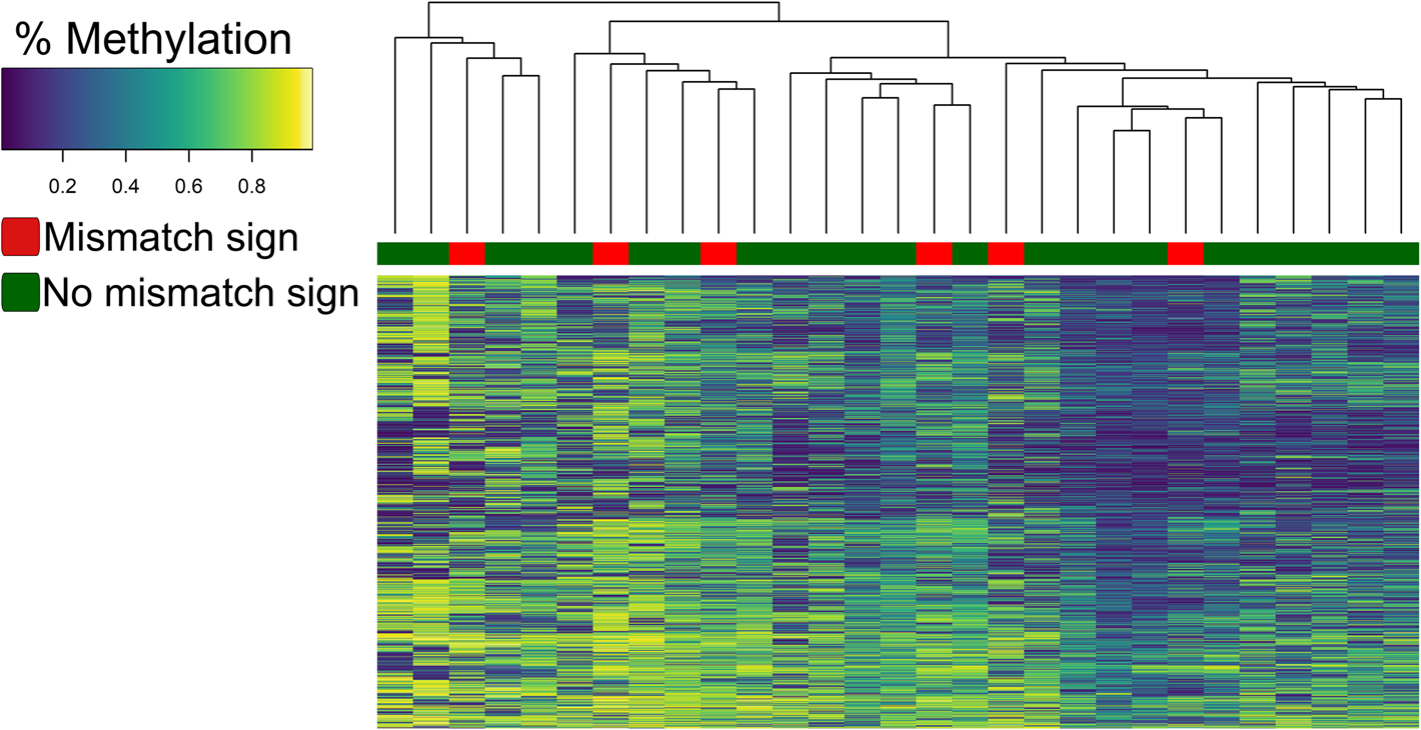
Unsupervised hierarchical clustering analysis on methylation levels for the 5000 most deviating CpG sites in the EPIC methylation array. A value of 0.2 indicated low percentage of methylation and 0,8 a high percentage of methylation. Clustering of the DNA-methylation profiles for the *IDH*-mut astrocytomas in the retrospective cohort (N = 29) did not identify a T2-FLAIR mismatch-methylation associated phenotype

DNA methylation profiling was performed for *IDH*-mut astrocytomas in the retrospective cohort where sufficient tumor tissue was available (*N* = 29) to determine of patients with a mismatch sign (*N* = 6) clustered together indicating a particular biological profile. Unsupervised hierarchical clustering with respect to the 5000 most deviating CpG sites in the methylation array grouped the *IDH*-mut astrocytomas into two main clusters as demonstrated in Fig. 6. However, patients with mismatch sign did not cluster together.

## Discussion

In this study there were no differences between *IDH*-mut astrocytomas with or without the mismatch sign with respect to extent of resection or any other clinical outcome parameter. Further, *IDH*-mut non-codel gliomas (astrocytomas) with mismatch sign did not harbor a unique methylation profile. The only difference we found between the patients with *IDH*-mut astrocytomas with mismatch sign and those without was related to the imaging finding itself. However, we validate that the T2-FLAIR mismatch sign has substantial interrater agreement and high specificity for *IDH*-mut astrocytomas. The clinical implications of mismatch sign are currently limited to this particular association.

During surgery it is evident that gliomas may vary in texture and appearance, and with the radiological image of homogenous signal on T2-weighted sequences and seemingly conspicuous tumor border, the question was raised whether the extent of the resection is related to the mismatch sign. This may be of particular importance, since the *IDH*-mut astrocytoma group seems to be the one benefitting most from extensive surgery [13, 14, 26]. In our cohort, the extent of resection did not differ between groups, hence mismatch sign should not be taken as a factor influencing extent of resection in *IDH*-mut astrocytomas. To our knowledge, this is the first study investigating the clinical factors and extent of resection in relation to the mismatch sign. One previous study evaluated the association between survival and the mismatch sign, with a median follow-up of 65.7 months, and found no differences in overall survival between groups [7]. This finding that mismatch sign does not indicate a particular prognostic group is further corroborated by our data.

Our results on interrater variability validate the data from Broen et al, who found a kappa value of 0.75, which is considered a substantial interrater agreement [17, 25]. According to the literature, a kappa value within the range of 0.56–0.79 is moderate to substantial [7, 17, 25, 27]. Thus, the collective experience so far is that the T2-FLAIR mismatch sign can reliably be detected in clinical practice [7, 17, 27].

It should be noted that we tested the diagnostic properties of the mismatch sign with different patient selections, which is a strength compared to a pure histopathological selection in previous studies [7, 12, 17]. In our selection based upon histopathology, we identified two patients with mismatch sign who had *IDH*-mut codel glioma, unlike previous studies by Broen et al and Patel et al who presented a 100% specificity for *IDH*-mut astrocytomas [7, 17]. However, later reports had made similar findings to ours, and the overall specificity reported in the literature is therefore in the range of 96.0–100.0% [7, 8, 12, 17, 28]. The mismatch sign has been found occasionally in *IDH*-mut codel gliomas, but also in pediatric low-grade brain tumors. This far, the mismatch sign has been reported in pilomyxoid astrocytoma, LGG harboring *MYB* rearrangement, oligodendroglioma (*IDH*-mut codel), and even in one patient with a non-neoplastic lesion [8].

In our selection of patients with radiologically suspected LGG other tumor diagnoses may also be encountered. Indeed, one patient in this prospective cohort with T2-FLAIR mismatch sign had an *IDH*-mut glioblastoma*,* suggesting that the mismatch sign is not grade specific. Importantly, there were no other differential diagnoses beyond diffuse gliomas that presented with the mismatch sign. Although of low sensitivity (27.1–51.0%), the specificity for *IDH*-mut astrocytomas renders the evaluation of mismatch sign useful in a clinical setting for individual cases [12, 17, 28, 29]. Adding advanced imaging parameters like apparent diffusion coefficient (ADC) and cerebral blood volume (CBV) to the mismatch sign may further improve the diagnostic capabilities of *IDH*-mut astrocytomas, although at the cost of increased complexity [30–33].

In an effort to understand the biological importance of the mismatch sign, we used DNA methylation analyses and unsupervised hierarchical clustering in a small subsample of patients with *IDH*-mut astrocytoma from the retrospective cohort. Clustering analysis could not distinguish between samples with mismatch sign from those without. Thus, this could indicate that the mismatch sign did not have a common overall methylation profile. The only other in-depth analysis of biology so far was performed by Patel *el al*, who found no convincing differences in biology, including methylation analysis [34]. Finally, since survival is consistently reported not to differ between groups with or without mismatch sign [7], it seems unlikely that the patients with mismatch sign constitute a specific type of *IDH*-mut astrocytomas.

### Strength and limitations

Strengths of this study include the both histopathological and image-based selection in the evaluation of the T2-FLAIR mismatch sign. Our prospective cohort of patients with suspected LGG reflects clinical neuro-oncology practice, where also other relevant diagnoses may be encountered at times. The small sample size is one limitation, especially for the methylation subsample analyses. For survival analyses, a longer follow-up would have been preferable, as a part of the cohort recently underwent surgical treatment. Since this was an exploratory study of clinical factors associated with the mismatch sign, we did not adjust for multiple comparisons and thereby increasing the chance of false positive associations simply by chance. However, we did not find *any* significant association, even without this adjustment.

## Conclusion

The T2-FLAIR mismatch sign in patients with *IDH*-mut astrocytomas was not found to be associated with clinical variables such as presenting symptoms, extent of resection, or survival. Methylation analysis further strengthens the previous indications that the *IDH*-mut astrocytomas with mismatch sign does not compromise a specific subentity. Finally, we validate the T2-FLAIR mismatch sign as a reliable marker with high specificity of *IDH*-mut astrocytomas, but with limited sensitivity.

## Supplementary information

**Additional file 1: Supplementary material.** “Data discordant cases T2-FLAIR mismatch sign”

**Additional file 2: Supplementary Fig. 1a-b**. I a) FLAIR sequence demonstrating a relative hypointense signal with the exception of a hyperintense peripheral rim. b) T2W sequence demonstrating homogenous hyperintensive signal with a conspicuous border. This glioma was considered to have a mismatch sign.

**Additional file 3: Supplementary Fig. 2a-b**. a) FLAIR sequence demonstrating a relative hypointense signal with the exception of a hyperintense peripheral rim. b) T2W sequence demonstrating homogenous hyperintensive signal with a conspicuous border. This glioma was considered to have a mismatch sign.

**Additional file 4: Supplementary Fig. 3a-b.** a) FLAIR sequence demonstrating hyperintensive signal with diffuse border. b) T2W sequence demonstrating hyperintensive signal with diffuse border. This glioma was considered not to have a mismatch sign.

## Data Availability

The datasets used and analyzed during the current study are available from the corresponding author on reasonable request.

## Abbreviations

ADC: Apparent diffusion coefficient
CBV: Cerebral blood volume
Codel: 1p19q codeleted
DNET: Dysembryoplastic neuroepithelial tumor
FLAIR: Fluid-attenuated inversion recovery
IDH1 and 2: Isocitrate dehydrogenase genes 1 and 2
IDH-mut: Isocitrate dehydrogenase gene mutation
LGG: Lower-grade gliomas, defined as WHO grade II and grade III diffuse gliomas
MDT: Multidisciplinary team
MRI: Magnetic resonance imaging
NPV: Negative predictive value
Non-codel: 1p19q non-codeleted
NPV: Negative predictive value
Mismatch sign: T2-FLAIR mismatch sign
