# Supplementary material for: The clinical significance of the T2-FLAIR mismatch sign in grade II and III gliomas: a population-based study
Source: BMC Cancer. 2020 May 20;20:450. doi: 10.1186/s12885-020-06951-w (PMC7238512; doi:10.1186/s12885-020-06951-w)
Supplement: Supplementary file 1 — Additional file 1: Supplementary material. “Data discordant cases T2-FLAIR mismatch sign” [file 12885_2020_6951_MOESM1_ESM.docx]

Supplementary data

**Figure S1**


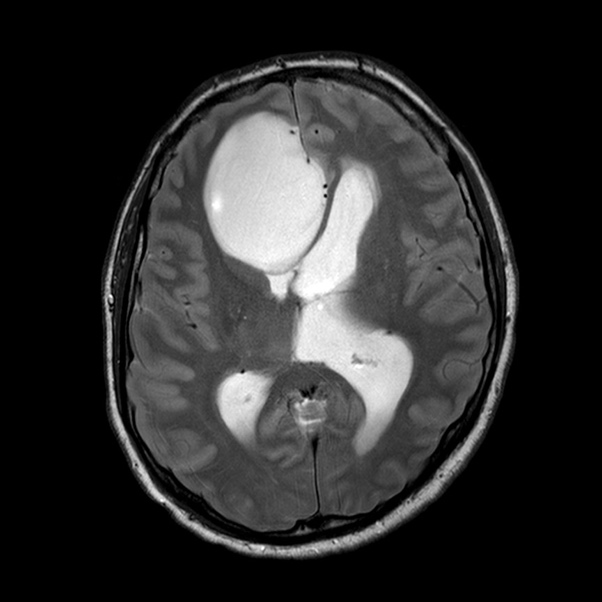

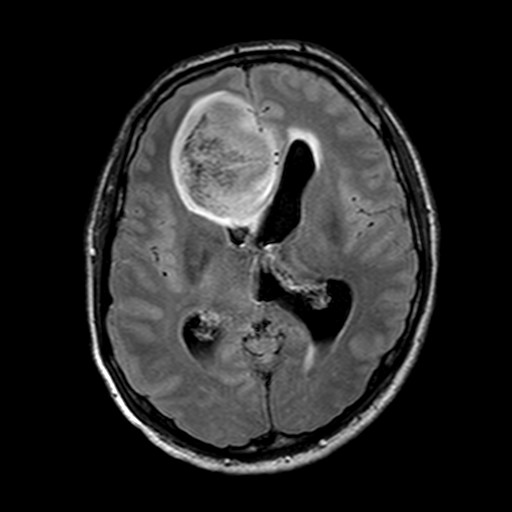


1a: FLAIR sequence 1b: T2 sequence

Diagnosis: Histological diagnosis of ganglioglioma grade II on primary surgery, on reoperation in 2013 diagnosed as astrocytoma grade III. *IDH*-mutated, and no 1p19q codeletion on methylation analysis.

Consensus review: Darker inner mass with hyperintensive peripheral rim on FLAIR, homogenous hyperintensive signal on T2. Mismatch sign present.

**Figure S2**


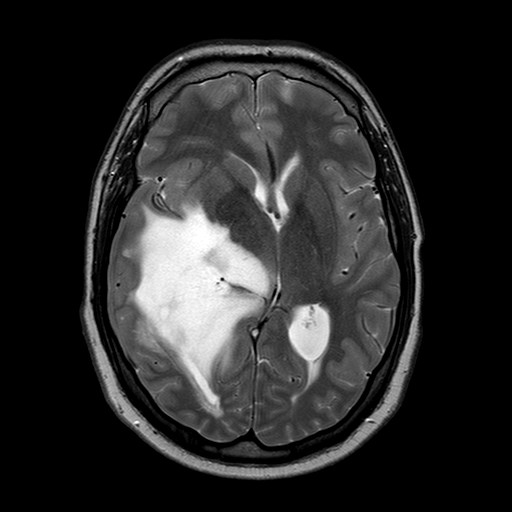

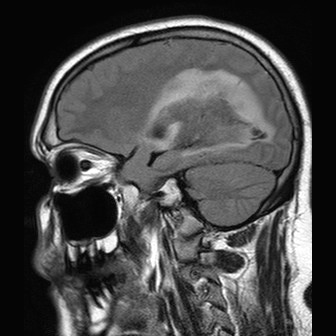


2a: FLAIR sequence, sagittal view 2b: T2 sequence

Diagnosis: Astrocytoma grade II. Status of *IDH*-mutation and 1p19q codeletion not analyzed.

Consensus review: Difficult case, edema but the peripheral rim is apparent and dark center with hyperintensive homogenous signal on T2. Mismatch sign present.

**Figure S3**


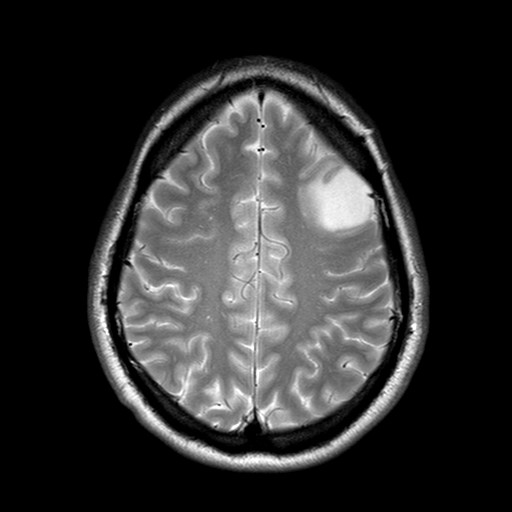

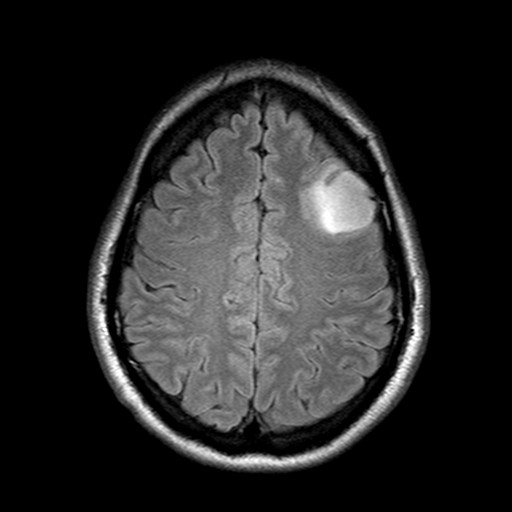


3a: FLAIR sequence 3b: T2 sequence

Diagnosis: Astrocytoma grade II, methylation analysis showed *IDH*-mutation and no 1p19q codeletion.

Consensus review: Lack of complete peripheral and apparent rim in FLAIR sequence and dark center. Conclusion, no mismatch sign present.

**Figure S4**


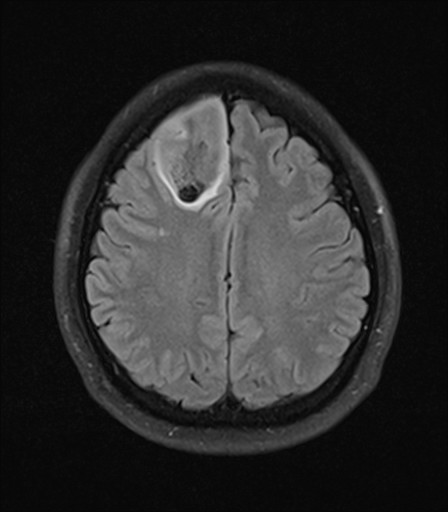


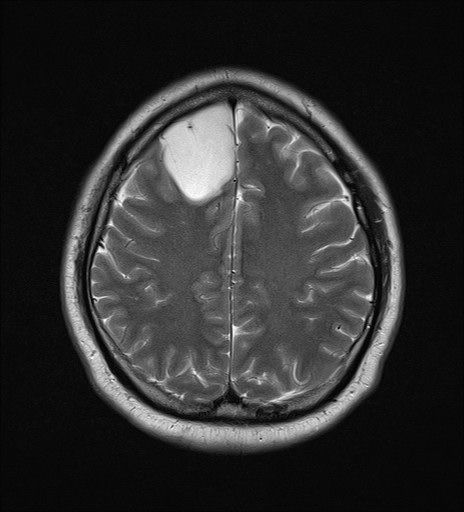


4a: FLAIR sequence 4b: T2 sequence

Diagnosis: Astrocytoma grade II. Status of *IDH*-mutation and 1p19q codeletion not analyzed.

Consensus review: Partially cystic, but with a distinct, hyperintensive rim and homogenous hyperintensive signal on T2. Mismatch sign present.

**Figure S5**


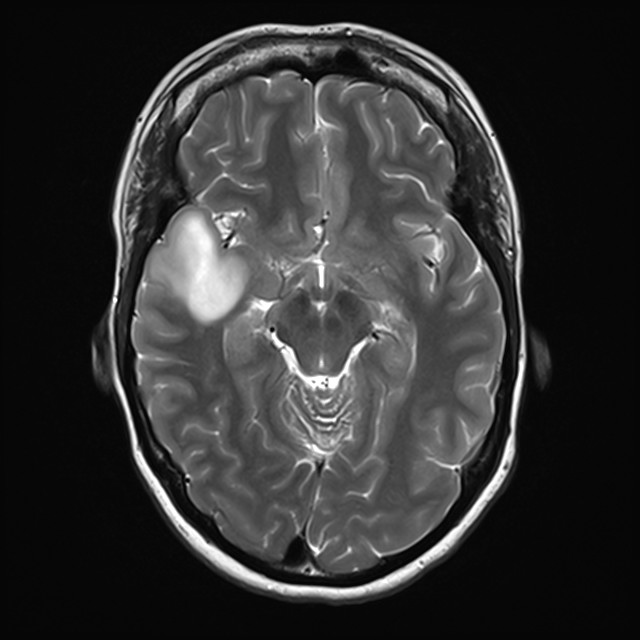

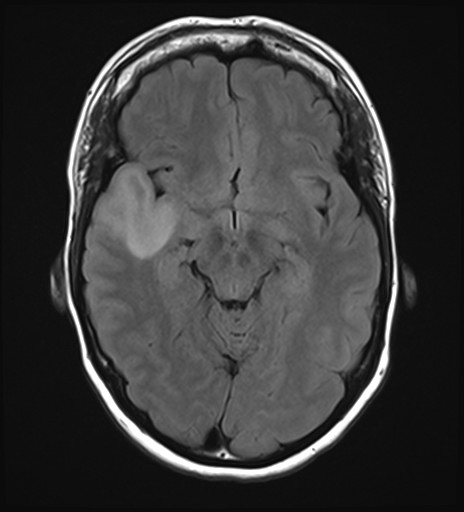


5a: FLAIR sequence 5b: T2 sequence

Diagnosis: Oligodendroglioma grade II, *IDH*-mutation and 1p19q codeletion

Consensus review: Lack of peripheral rim and homogenous hyperintensive signal on T2. No mismatch sign present.

**Figure S6**

**
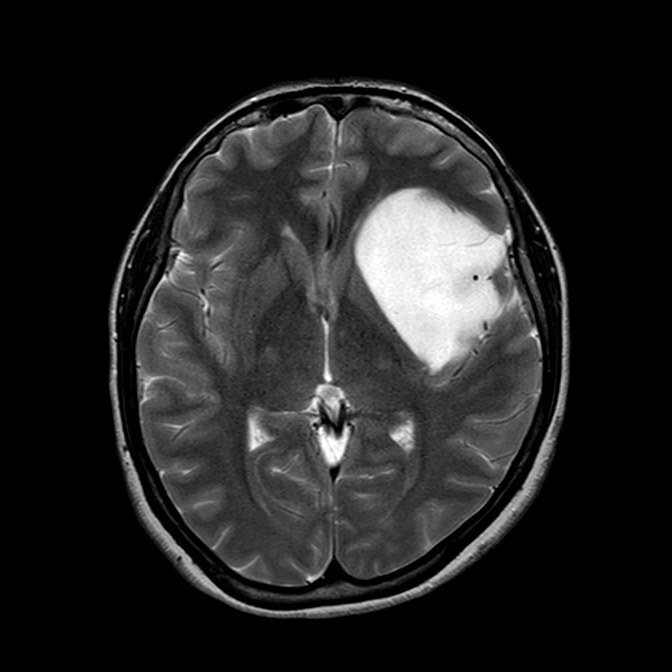

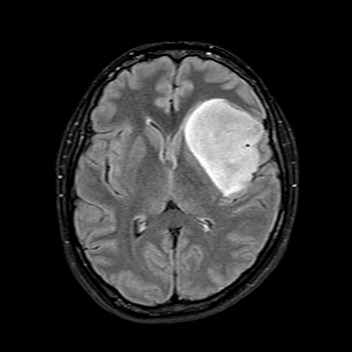
**

6a: FLAIR sequence 6b: T2 sequence

Diagnosis: Astrocytoma grade II, *IDH-*mutation and no 1p19q codeletion.

Consensus review: Too bright center on FLAIR, perhaps peripheral ring but too high intensity on the center. No mismatch sign present.


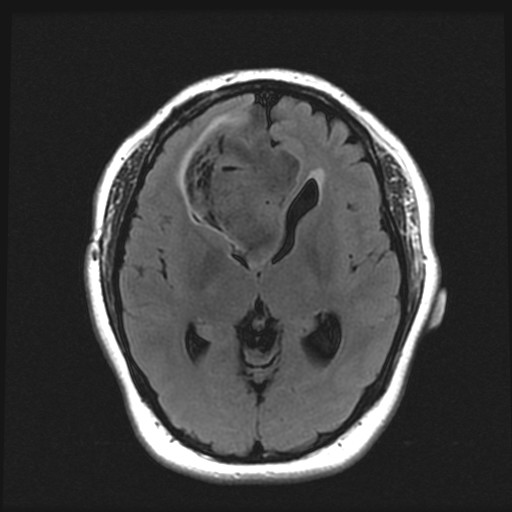
**Figure S7**


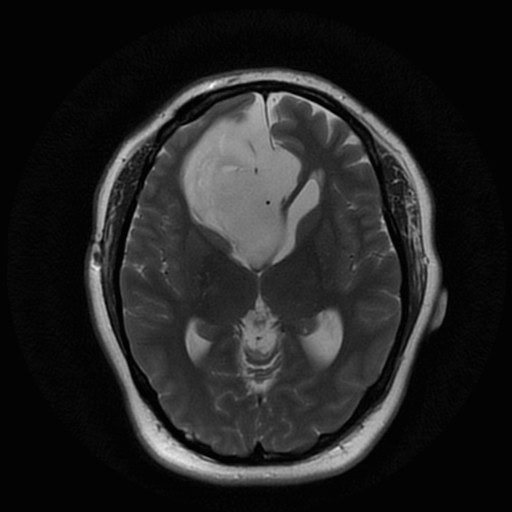


7a: FLAIR sequence 7b: T2 sequence

Diagnosis: Astrocytoma grade II, *IDH*-mutation and no 1p19q codeletion.

Consensus review: Smaller cystic components and only partial rim on FLAIR but still significant mismatch. Mismatch sign present.

**Figure S8**


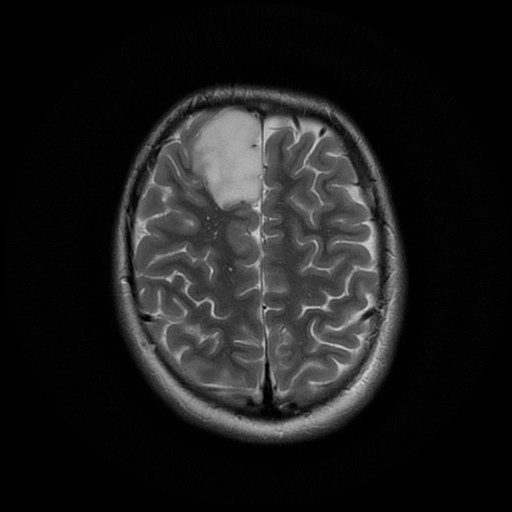

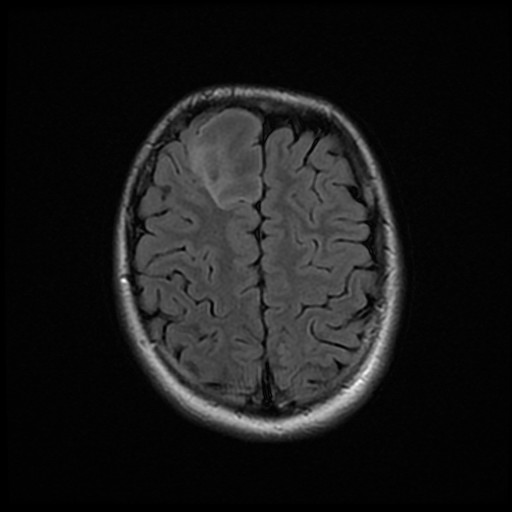


8a: FLAIR sequence 8b: T2 sequence

Diagnosis: Astrocytoma grade II, *IDH*-mutation and no 1p19q codeletion.

Consensus review: Very difficult case, heterogenous structures on T2. No mismatch sign present.

**Figure S9**


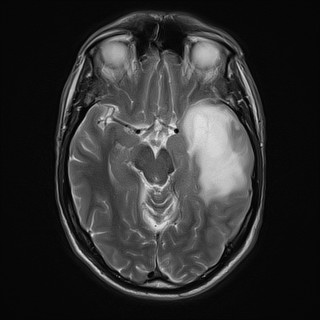


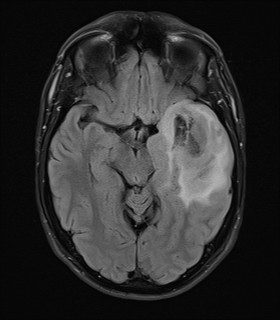


9a: FLAIR sequence 9b: T2 sequence

Diagnosis: Astrocytoma grade II, *IDH*-mutation and no 1p19q codeletion.

Consensus review: Edema which causes difficulties in assessment, lack of obvious peripheral rim, heterogenous signal on T2. No mismatch sign present.
