# Supplementary figures and images for: The clinical significance of the T2-FLAIR mismatch sign in grade II and III gliomas: a population-based study
Source: BMC Cancer. 2020 May 20;20:450. doi: 10.1186/s12885-020-06951-w (PMC7238512; doi:10.1186/s12885-020-06951-w)

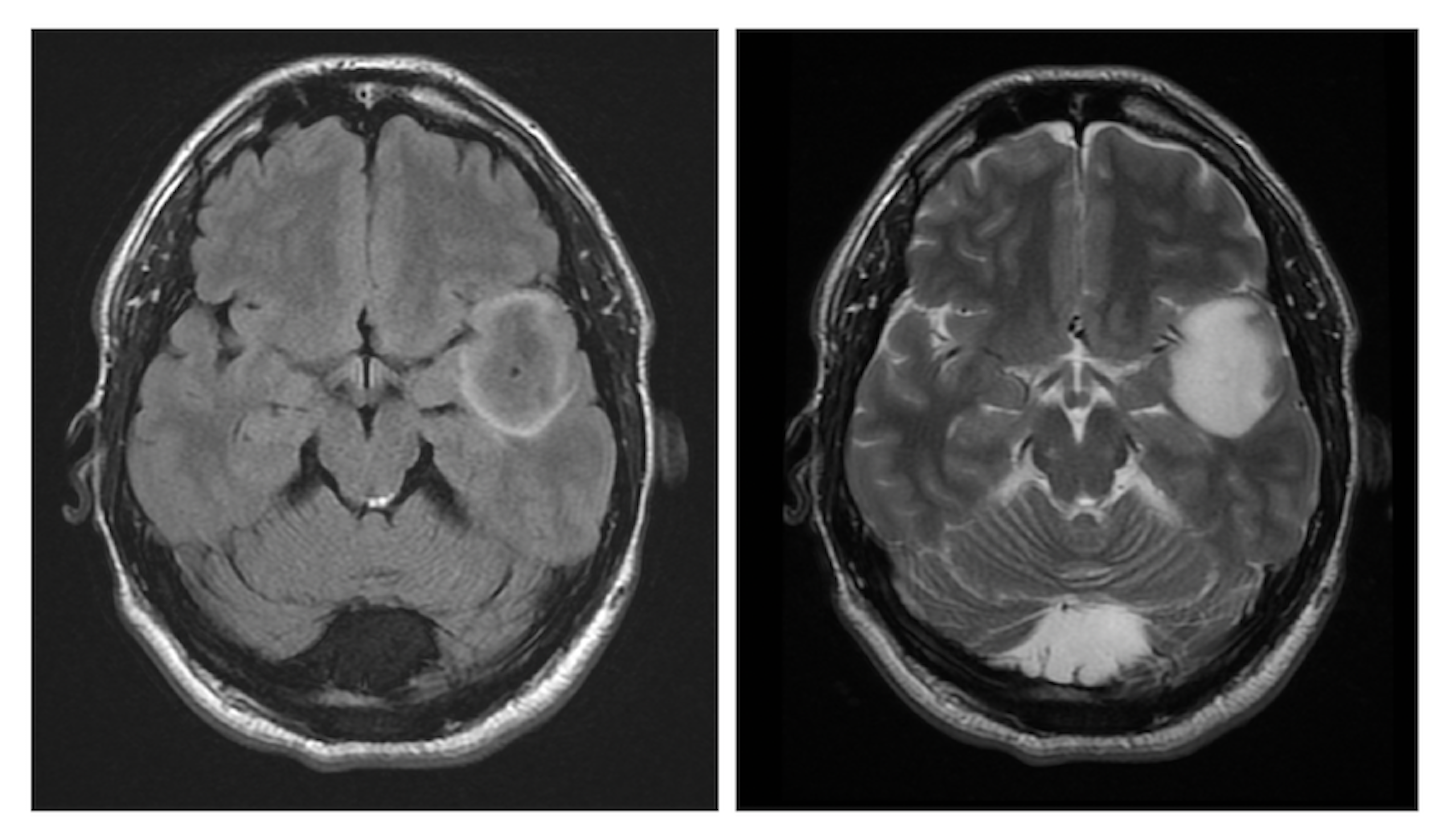

Supplement: Supplementary file 2 — Additional file 2: Supplementary Fig. 1a-b. I a) FLAIR sequence demonstrating a relative hypointense signal with the exception of a hyperintense peripheral rim. b) T2W sequence demonstrating homogenous hyperintensive signal with a conspicuous border. This glioma was considered to have a mismatch sign. [file 12885_2020_6951_MOESM2_ESM.tiff]

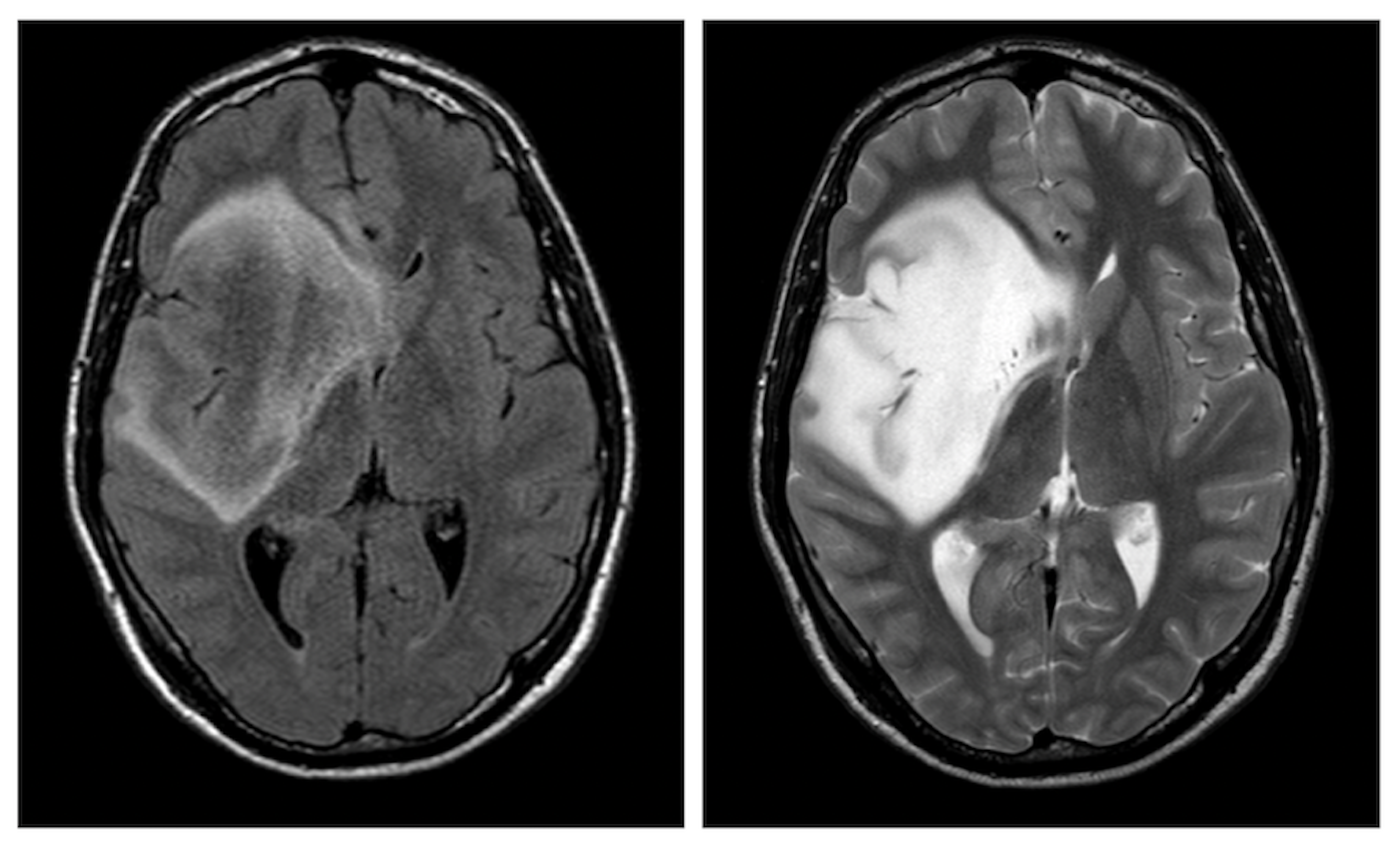

Supplement: Supplementary file 3 — Additional file 3: Supplementary Fig. 2a-b. a) FLAIR sequence demonstrating a relative hypointense signal with the exception of a hyperintense peripheral rim. b) T2W sequence demonstrating homogenous hyperintensive signal with a conspicuous border. This glioma was considered to have a mismatch sign. [file 12885_2020_6951_MOESM3_ESM.tiff]

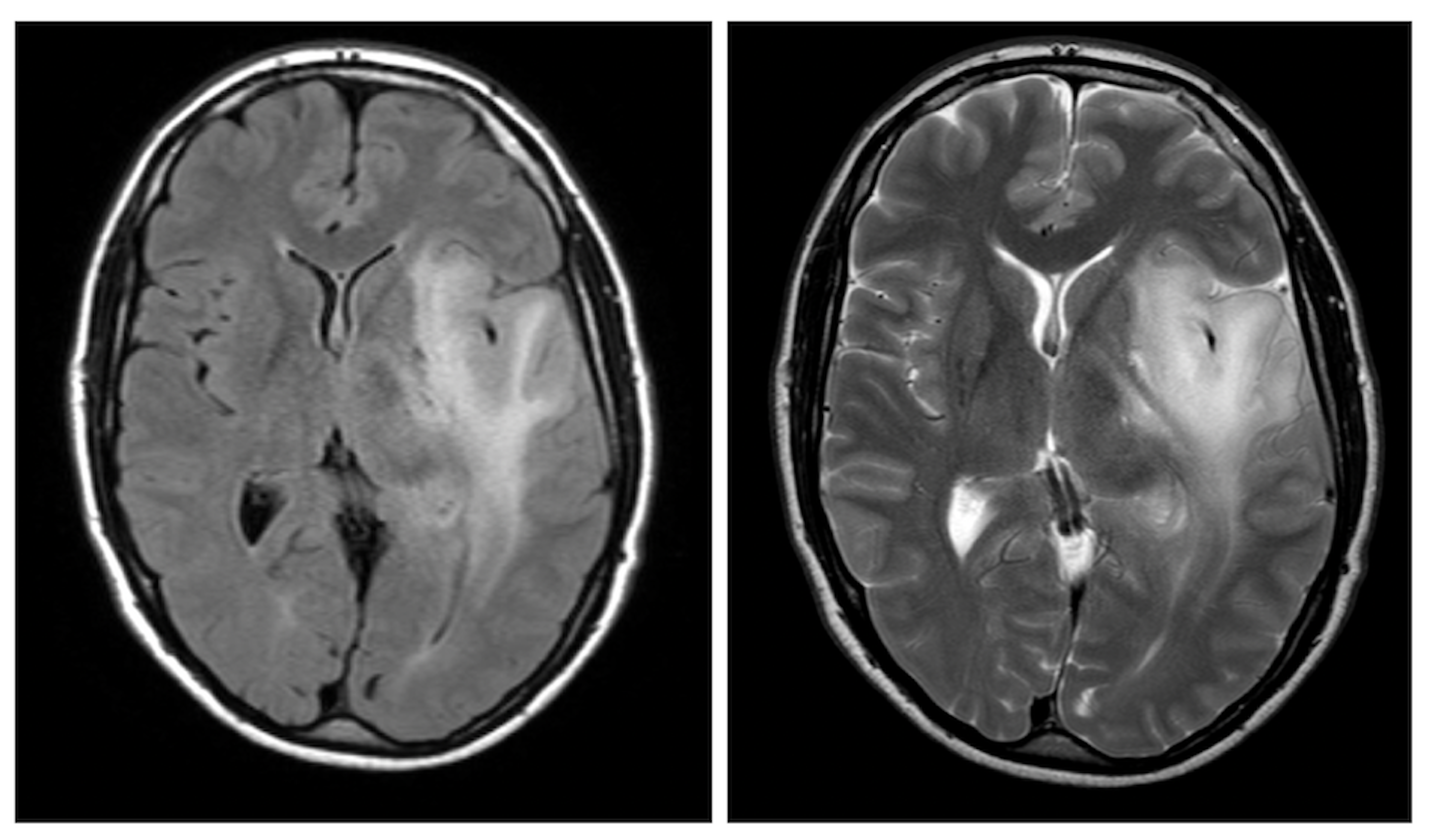

Supplement: Supplementary file 4 — Additional file 4: Supplementary Fig. 3a-b. a) FLAIR sequence demonstrating hyperintensive signal with diffuse border. b) T2W sequence demonstrating hyperintensive signal with diffuse border. This glioma was considered not to have a mismatch sign. [file 12885_2020_6951_MOESM4_ESM.tiff]
